# Immunological consequences of microbiome-based therapeutics

**DOI:** 10.3389/fimmu.2022.1046472

**Published:** 2023-01-12

**Authors:** Md Zahidul Alam, Jeffrey R. Maslanka, Michael C. Abt

**Affiliations:** Department of Microbiology, Perelman School of Medicine, University of Pennsylvania, Philadelphia, PA, United States

**Keywords:** mucosal immunity, regulatory T cells, fecal microbiota transplantation, probiotics, live biotherapeutic products, *C. difficile*, microbiome-based therapeutic, immune checkpoint inhibitors

## Abstract

The complex network of microscopic organisms living on and within humans, collectively referred to as the microbiome, produce wide array of biologically active molecules that shape our health. Disruption of the microbiome is associated with susceptibility to a range of diseases such as cancer, diabetes, allergy, obesity, and infection. A new series of next-generation microbiome-based therapies are being developed to treat these diseases by transplanting bacteria or bacterial-derived byproducts into a diseased individual to reset the recipient’s microbiome and restore health. Microbiome transplantation therapy is still in its early stages of being a routine treatment option and, with a few notable exceptions, has had limited success in clinical trials. In this review, we highlight the successes and challenges of implementing these therapies to treat disease with a focus on interactions between the immune system and microbiome-based therapeutics. The immune activation status of the microbiome transplant recipient prior to transplantation has an important role in supporting bacterial engraftment. Following engraftment, microbiome transplant derived signals can modulate immune function to ameliorate disease. As novel microbiome-based therapeutics are developed, consideration of how the transplants will interact with the immune system will be a key factor in determining whether the microbiome-based transplant elicits its intended therapeutic effect.

## Section 1: Introduction. Microbiome-based therapy and immune health

Millions of years of co-evolution between microbiome and the mammalian host has resulted in mutualism and homeostasis (1). Microbial diversity is a hallmark feature of a healthy microbiome as the trillions of microbes act in concert to support host health (2, 3). However, this mutualistic relationship can be breached by environmental factors, such as antibiotic use or dietary changes, that lead to decreased bacterial diversity or skewed relative abundance of bacterial species (4–6). Collectively, these changes in microbiome composition are referred to as microbial dysbiosis (7). Consistently, altered or ‘dysbiotic’ intestinal microbial communities are associated with disease states such as cancer, diabetes, allergy, obesity, neurological disorders, and infectious diseases (8–15). These associations have been replicated in animal studies, leading to increased mechanistic understanding of how microbial dysbiosis can drive disease progression. Numerous observations linking dysbiosis and disease have provoked the biomedical community to hypothesize that resetting the microbiome with a composition of bacteria from healthy individuals could restore health (16–18). In support of this theory, numerous animal studies demonstrate resolution of disease following microbiome transplantation directly into the host’s intestine (19–24). In humans, however, the efficacy of microbiome-based therapies to treat disease have been inconsistent (25). The complex challenge of developing successful microbiome-based therapies is exemplified by the multifaceted, bidirectional interactions between commensal bacteria and the host immune system (7).

At steady-state, intestinal bacteria are critical regulators of the host immune system that promote immune education, immunotolerance, as well as resistance to pathogens (15). For example, distinct species of the endogenous microbiome directly impact CD4^+^ T helper (T_H_) cell differentiation and function in the intestine. Resident segmented filamentous bacteria (SFB) attach to the small intestinal epithelium and drive CD4^+^ T_H_17 cell differentiation that is essential to maintain the integrity of the intestinal barrier (26, 27). SFB colonization induce antigen-specific T_H_17 cells in the small intestine *via* IL-22, IL-23, and serum amyloid A protein (SAA) protein and promote host defenses against enteric bacterial pathogens through CD4^+^ T cell dependent and independent mechanisms (26, 28, 29). Similarly, *Bacteroides fragilis* contributes to the differentiation of T_H_1 cells and lymphoid organogenesis by its capsular polysaccharide A (PSA). Colonization of germ-free mice with PSA produced from *B. fragilis* restores splenic IFN-γ producing CD4^+^ T cells through antigen presentation in an IL-12/STAT4 specific manner (30). Further, *B. fragilis* helps to maintain T_H_1/T_H_2 balance and directs the generation of the CD4^+^ regulatory T cells (T_reg_) *via* PSA (30, 31). Firmicutes, specifically bacteria belong to the Clostridia class, also promote immune homeostasis at steady-state by promoting T_reg_ cell differentiation in the intestine *via* production of short chain fatty acids (SCFA) and secondary bile acids (32–39). Insights gained from these studies as well as many others (40, 41) that investigate immune-microbiome interactions at steady-state have laid the foundation to use the microbiome as a therapeutic that can be administered to an individual to recalibrate the immune system and treat disease.

The collective microbiome has been referenced as a distinct organ system, comparable to the kidney or liver organ, due to the capacity of the microbiome to synthesize signaling molecules that shape host physiology (42). A dysbiotic microbiome that fails to function properly is analogous to an organ system failure (43). Likewise, a microbiome-based transplant can be considered a bacterial “organ” transplant meant to replace the dysbiotic “failing organ” with a healthy one. As with kidney transplants, there are complex molecular interactions between the donor microbiome “organ” and the host that will determine whether the transplant successfully engrafts or is rejected. Crosstalk between the microbiome transplant and the host immune system has an important role in determining transplant success or failure and is the focus of this review.

## Section 2: Dysbiosis and disease

It is often challenging to determine whether a dysbiotic intestinal microbiome is the cause or the consequence of the disease state. Chronic intestinal dysbiosis is associated with elevated expression of proinflammatory mediators that are hallmarks of various chronic inflammatory diseases (7, 41). For example, the intestinal microbiome in patients with inflammatory bowel disease (IBD) is characterized by a significant decrease in Firmicutes bacteria and an increase in Bacteroidetes and Proteobacteria (44). Instead of a diverse, Firmicutes-enriched microbiome that promotes intestinal T_reg_ cell population and immune tolerance (34, 35, 45), the dysbiotic microbiome in IBD patients supports pathogenic CD4^+^ type-17 T helper (T_H_17) cells in the intestine. In contrast to the T_H_17 cell population induced by commensal bacteria that maintain barrier integrity in the context of a diverse microbiome, T_H_17 cells that arise under dysbiotic conditions release pro-inflammatory cytokines, causing intestinal damage (46–48). Inflammatory T_H_17 cells accumulate in IBD patients’ intestinal lesions both in the ileum and colon (49, 50). Several studies support that the dysbiotic intestinal microbiome in IBD is the causative agent that initiates immune changes in the colon. For instance, Britton et al. show that germ-free mice transplanted with the microbiome obtained from IBD patients had increased numbers of T_H_17 and T_H_2 cells and reduced RORγt^+^ T_reg_ cells in their colon compared to germ-free mice that received microbiome from healthy donors (51). Using a T cell transfer model of colitis this group demonstrated the IBD microbiome caused more severe CD4^+^ T cell-mediated colitis, compared to healthy microbiome (51). Dysbiosis caused by external perturbation, such as antibiotic treatment, can also lead to activation of host inflammatory mediators, that provide feedback to the microbiome and drive expansion of potentially pathogenic intestinal bacteria. Byndloss et al. found that antibiotic-mediated depletion of butyrate-producing bacteria reduced peroxisome proliferator–activated receptor gamma (PPAR-γ) expression in the intestinal epithelium, leading to the induction of NOS2 and increased the nitrate levels in the colon (52). Host-derived nitrate can be used as respiratory electron acceptors by colitogenic *Escherichia/Shigella* or invasive *Samonella* bacteria in the intestine (53, 54). In both examples, the antibiotic-induced dysbiotic microbiome led to breakdown of intestinal immune homeostasis that leaves the host more vulnerable to colonization with disease-promoting bacteria.

Reciprocal to dysbiosis driving aberrant immune responses, host inflammation can cause intestinal dysbiosis. Several studies show that excessive intestinal inflammation due to infection or IBD can alter the intestinal lumen microbial population (55, 56). As a consequence of intestinal mucosal inflammation, inflammatory by-products like reactive oxygen species and reactive nitrogen species derivatives can change the intestinal microbiome by providing a growth advantage to inflammation-tolerant colitogenic bacteria (e.g., *Gammaproteobacteria*) over the inflammation-sensitive (e.g., *Bifidobacteria*) beneficial bacteria (53, 57, 58). For instance, infection due to *Toxoplasma gondii*, causes a marked immune-driven intestinal dysbiosis by increasing macrophage-derived NO byproducts in the colon that supports anaerobic respiration in *E. coli* (59). Likewise, in recurrent *Clostridioides difficile* infection patients, increased intestinal inflammation is associated with an abundance of *Escherichia/Shigella* in the intestinal tract (60, 61). Studies using genetically modified mice or mice treated with immunostimulatory agents to initiate immune activation demonstrate that immune activity can result in dysbiosis. Dysbiosis that is caused by immune activation renders the host more susceptible to severe colitis (62–64). Combined, these studies demonstrate the bidirectional influence of immune system and dysbiosis.

In dysbiosis-associated diseases, it is difficult to determine whether the altered microbiome is driving pathogenic immune responses or, conversely, dysregulated immunity promotes microbial dysbiosis. In reality, a reciprocal relationship exists where both the dysbiotic microbiome and aberrant immune system feed forward to reinforce dysbiosis and immunopathology. Microbiome-based therapies seek to break this cycle by resetting the microbiome back to a “healthy” state. The immunologic consequences of such therapies need to be considered for successful implementation of such therapies.

## Section 3: Types of microbiome-based therapies

Microbiome-based therapeutics seek to restore the healthy microbial populations that reside on or within the host and the downstream metabolic networks that the microbiome directs. Broadly, microbiome-based therapies can be categorized into five approaches. These are prebiotics, fecal microbiota transplantation (FMT), probiotics, live-biotherapeutics, and postbiotics (**Table 1**).

**Table 1:** Categories of microbiome-based therapies.

Therapeutic	Description	Interaction with immune system	Ref
Prebiotics (e.g Galacto-oligosaccharides)	Non-digestible molecules to boost bacterial growth	Reduced allergic T_H_2 responses Improved NK cell function	(65, 66)
Fecal Microbiota Transplantation	Feces from a healthy donor	Increased T_reg_ cells, decreased T_H_1 cells	(67, 68)
Probiotics (e.g. *Bifidobacterium)*	Live non-permenant resident microorganism,	Increased T_reg_ cells Anti-inflammatory in colitis models	(69–71)
Live bio-therapeutics (e.g. SER-287)	Select, live microorganism(s)provide clinical benefit	Increased T_reg_ cell abundance and function	(38, 72–74)
Postbiotics (e.g. Butyrate)	Bacterial metabolites or bacterial components	Increased T_reg_ cells, decreased T_H17_ cells Reduced innate cell sensitivity to microbiome Decreased infiltration of colonic neutrophils and lymphocytes	(34, 36, 75, 76)

### 3.1 Prebiotics

Prebiotics are non-living food components that are not digestible by the host but beneficially impact the host’s health by selectively stimulating the activity and growth of some genera of microbes in the intestine (65, 77). Current prebiotics are predominantly carbohydrate-based, but other substances, such as polyunsaturated fatty acids and polyphenols, also exert prebiotic effects (65). An ideal prebiotic can resist the actions of acids, bile salts, and other hydrolyzing enzymes in the intestine, is non-absorbable in the upper gastrointestinal tract, and can be fermented by beneficial intestinal microbes Fecal microbiota transplantation (FMT) (77). Among many prebiotics, non-digestible oligosaccharides, such as inulin and its hydrolysis products oligofructose, and (trans) galacto-oligosaccharides fulfill all those criteria (65, 66). Prebiotics prevent pathogen growth by increasing intestinal organic acids thereby reducing luminal pH levels and establishing a stable population of commensal bacteria that compete with invading pathogens for nutrients (78–80). Efficacy of prebiotic therapy, however, is dependent on beneficial microbes already residing within the host, a condition that may not be met in dysbiosis. Therefore, more direct microbiome-based therapeutic modalities may be needed.

### 3.2 Fecal microbiota transplantation

FMT, also known as bacteriotherapy, is a procedure to obtain feces from a healthy individual and introduce the fecal slurry into a patient’s intestinal tract. FMT can restore the recipient’s gastrointestinal bacterial diversity and bacterial-derived metabolites termed the metabolome (81, 82). FMT is considered the crudest form of live microbiome based therapies since the transplant inoculum is often an undefined mixture of microbes. The first known description of the therapeutic use of feces was by Ge Hong for the treatment of human diseases, including diarrhea, in the fourth century in China (83). However, it was not until the mid-twentieth century that feces were used to treat a specific disease in a controlled way. In 1958, Eisenmen and colleagues were the first to treat pseudomembranous colitis with FMT (84). More recently, FMT to treat recurrent *C. difficile* infection is the most widely used application of this microbiome-based therapeutic. In controlled, double-blinded studies, FMT has been proven to be ~89% efficacious in preventing recurrent *C. difficile* infection (85). During *C. difficile* infection there is a decrease in SCFAs and secondary bile acids in patients’ colons (60, 86). Following FMT, the clinical improvement in patients is directly associated with engraftment of donor microbes, restoration of SCFAs and secondary bile acids, and resolution of inflammation in the colon (82, 87, 88). Bacteria to bacteria interactions and restoration of commensal bacteria colonization resistance are thought to be the primary FMT mechanism of action for resolution of *C. difficile* infection. In agreement with this prevailing theory, SCFAs and secondary bile acid metabolites can directly inhibit *C. difficile* growth (89, 90). However, as described in the following sections, FMT may also work indirectly *via* signaling with the host immune system.

FMT is also being studied for the treatment of non-infectious intestinal diseases like IBD (91) as well as extraintestinal diseases such as immune checkpoint inhibitor (ICI) cancer immunotherapies (92), metabolic, and neuropsychiatric disorders (93). Clinical trials using FMT to treat these diseases have more varied success compared to the use of FMT to treat recurrent *C. difficile* infection. In a systematic meta-analysis assessing the efficacy of FMT for the treatment of ulcerative colitis (UC), Narula et al. found that 28% of patients progressed to clinical remission of active UC following FMT. This was a marked improvement over the 9% remission in the placebo groups but still demonstrated that the majority of FMT recipients did not experience a therapeutic benefit from the FMT (94). Both animal and human trials with FMT have demonstrated the feasibility but also potential dangers of using an undefined bacterial consortium as a therapy (95). This concern has led to slow adoption of crude FMT as a standard practice and search for more refined forms of FMT. A clean mixture of purely isolated gut bacteria collected from a healthy donor termed ‘defined gut microbial ecosystem components’- effectively cleared *C. difficile* from two patients who failed to clear the pathogen with several courses of traditional antibiotics therapy (96, 97). This defined FMT approach may be a safer, more controlled, and more acceptable method. However, future large-scale randomized controlled trials are needed to determine the efficacy of this defined FMT approach. Next generation microbiome-based therapies that are described below seek to further refine and remove donor variability inherent with FMT.

### 3.3 Probiotics

Probiotics are live microorganisms, mainly *Lactobacillus*, *Bifidobacterium* and *Saccharomyces*, that do not naturally reside in the mammalian host but provide health benefits when administered (98). The discovery that fermented food such as yogurt and fermented milk contain mixtures of bacteria with health benefits led to the development of modern probiotics. These bacteria can be isolated, grown, and administered in controlled doses. Probiotics can provide health benefits through several mechanisms including producing antimicrobial peptides, enhancing mucus production, and regulating mucosal immune functions (65). Several studies show an anti-inflammatory role of probiotics in alleviating colitis in mouse model (69–71). Although promising in animal studies, the therapeutic effect of probiotics in treating inflammatory diseases in patients is not well established. *Lactobacillus* and *Bifidobacterium* probiotic treatment failed to reduce TNF-α or increase IL-10 expression in IBD patients (99). However, several clinical studies demonstrate that combinations of probiotics provide some benefit in UC patients (100). A systematic review with a meta-analysis by Derwa et al. that included 22 randomized controlled trials that administered probiotics to IBD patients demonstrated no therapeutic benefit of probiotics at inducing remission of active UC compared to the placebo group (101). In the same meta-analysis, the probiotics effect was similar to 5-aminosalicylic acid (5-ASA), a commonly used anti-inflammatory drug, in preventing UC relapse. In addition, no benefit of probiotics was observed in inducing remission of active Crohn’s disease (CD) as well as in preventing relapse of quiescent CD. In *C. difficile* infection, administration of the probiotic, *Saccharomyces boulardii* showed promise in preventing recurrence. McFarland and colleagues (102) found that oral administration of *S. boulardii* for four weeks with standard antibiotics therapy significantly reduced recurrent *C. difficile*-associated disease (34.6%), compared to the placebo group (64.7%). In another study (103), *S. boulardii* was effective in decreasing recurrences (16.7%) only when administered with a high-dose of vancomycin (2 grams/day), compared to placebo with a high-dose-vancomycin group (50%). In both studies, *S. boulardii* was effective with concurrent antibiotic therapy. The limitation of probiotic therapy could be due to the inability of the probiotic bacteria to colonize and take up residence in the dysbiotic intestinal tract and stably restore microbiome diversity.

### 3.4 Live bio-therapeutics

Distinct from probiotics, live bio-therapeutics are a single bacterial species or a selected combination of bacteria, that can colonize the intestine and are designed to provide a clinical benefit for a particular disease (104). Live bio-therapeutics are often identified and isolated from fecal bacterial populations. They are considered “next-generation” microbiome therapy products to treat diseases like *C. difficile* and IBD with more refinement than a heterogeneous fecal transplant mixture. A series of encouraging clinical trials have demonstrated the potential of this more refined microbiome-based therapy approach. A consortium of purified Firmicutes spores developed by Seres Therapeutics was effective in a phase 3 clinical trial to reduce the risk of *C. difficile* recurrence compared to standard of care antibiotics (105). Further, a defined collection of 8 bacteria, named VE303, developed by Vedanta Biosciences also shows promise in phase 2 clinical trial for the treatment of recurrent *C. difficile* infection (88). Similarly, SER-287, an oral formulation of Firmicutes spores, showed promising results in a phase 1 clinical trial for the remission of mild to moderate UC (106). Due to the complexity of microbial interactions with the host, identifying a single or small combination of bacterial species that convey comparable therapeutic effects to FMT is challenging. However, the studies described above demonstrate that live bio-therapeutics in development have the capacity to treat a broad range of dysbiosis associated diseases and could be used as a safer alternative to FMT.

### 3.5 Postbiotics

Postbiotics are soluble components of microbial cells or their derived metabolites that can provide therapeutic benefits (107). SCFAs and secondary bile acids are two examples of well-studied microbial-derived metabolites being actively tested for therapeutic effects. SCFAs consist of a class of molecules produced during bacterial fermentation of complex fibers in the colon (108). Administration of SCFAs reduces colitis in mice (45, 109), however, human IBD trials show inconsistent results (110). Further, secondary bile acids show anti-inflammatory effects on immune cells and alleviate intestinal inflammation in a mouse model of colitis (111). A clinical trial on the therapeutic effect of secondary bile acid, ursodeoxycholic acid in IBD is ongoing (**NCT03724175**). Notably, high physiologic concentrations of secondary bile acids drive colorectal tumorigenesis through oxidative stress and DNA damage in the epithelium (112, 113). The goal of postbiotics therapy is to identify and isolate downstream effector metabolites produced by the beneficial bacteria of the microbiome and administer these molecules to treat disease without the detrimental side effects. Postbiotic therapeutics offer the potential for chemical modifications to reduce off-target interactions and enhance on-target therapeutic effects.

## Section 4: The role of the immune system in shaping the outcome of microbiome-based therapy

**Figure 1:**
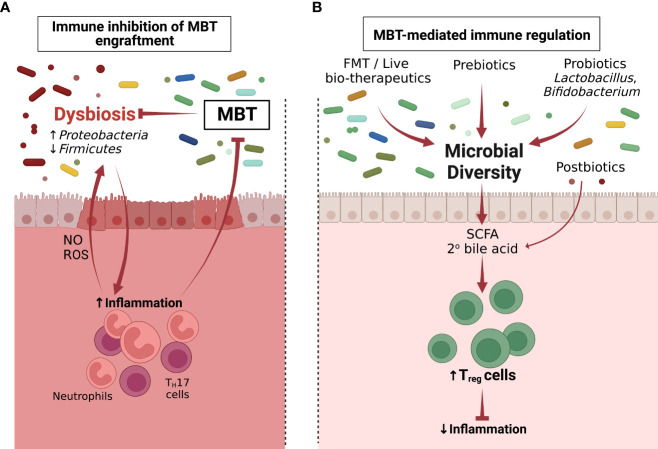
Microbiome-immune cell interactions determine the failure and success of microbiome-based therapies (MBTs). **(A)** Inflammation associated with dysbiosis renders the intestinal environment refractory to MBT engraftment (Section 4). **(B)** MBT can promote therapeutic effects through immune regulation following administration (Section 5). Following successful MBT engraftment, the overall microbiome diversity increases. Postbiotics or metabolites produced by incoming bacteria including SCFAs and 2° bile acids can induce CD4^+^ T_reg_ cells, which reduce inflammation and restore the immune homeostasis of the colon.

The immune status of the recipient prior to microbiome transplantation shapes the engraftment, function and ultimately efficacy of any microbiome therapy to treat disease (**Figure 1**). The microbiome transplant recipient’s immune status can be impacted by infection, genetics or pre-existing dysbiosis and in turn shape the intestinal microenvironment into a hostile or receptive setting for the microbial consortium attempting to colonize the host. Work from our group has begun to identify the immune components prior to microbiome therapy that determine whether the microbiome transplant is successful. In a mouse model of FMT-mediated resolution of *C. difficile* infection, FMT recipient *Rag1* ^-/-^ mice, which lack B and T cells, failed to engraft the transplanted bacteria, did not restore cecal secondary bile acid levels, and were not able to resolve *C. difficile* infection compared to immunocompetent littermate control mice (114). CD4^+^ T cells, specifically T_reg_ cells, were the critical adaptive immune cell type that supported FMT success as depletion of T_reg_ cells rendered *C. difficile* infected mice non-responsive to FMT therapy. Notably, uninfected *Rag1* ^-/-^ mice readily engraft the FMT bacterial consortium indicating that immune deficiency in combination with infection-driven inflammation is both needed to inhibit FMT engraftment. The intestinal T_reg_ cell compartment is comprised of heterogenous subsets of thymic-derived and peripherally-induced T_reg_ cells (115–118). These T_reg_ cell subsets promote tissue homeostasis mediated through a diverse repertoire of anti-inflammatory mechanisms, such as secretion of IL-10 and TGF-β, sequestration of IL-2, and expression of co-inhibitory molecules CTLA-4, ICOS and PD-1. In the absence of T_reg_ cell-mediated regulation, the intestinal environment can shift towards an inflammatory setting that results in elevated expression of pro-inflammatory mediators that provide a growth advantage to inflammation-tolerant bacterial species, and may skew microbial engraftment to favor these bacterial species over donor-derived, inflammation-sensitive bacteria (54, 119, 120). While it is known that inflammation can favor selective growth of some bacteria (54) further study is needed to identify which specific molecular pathways can be targeted to enable bacterial species from a microbiome transplant to engraft.

Clinical studies assessing the host inflammatory status prior to FMT further highlight the importance of immune status in transplant engraftment and thereby efficacy of such therapies. For example, recurrent *C. difficile* patients that have higher intestinal inflammation prior FMT, as measured by fecal calprotectin level, are more likely to require repeated FMT administration to resolve infection (121). Further, patients with recurrent *C. difficile* infection that have underlying IBD experience reduced beneficial effects following FMT therapy (122). Microbiome-based treatment of recurrent *C. difficile* infection in patients with underlying IBD is a difficult clinical challenge since both diseases have the potential to amplify mucosal barrier breakdown and dysregulate mucosal immunity. In a large retrospective study that used clinical metadata and colonoscopy to diagnosis patients’ IBD status, 74.4% of patients with concomitant IBD resolved *C. difficile* at 2 months following a single infusion of FMT compared to 92.1% of patients who did not have IBD (122). More evidence that pre-existing immune-driven inflammation impairs FMT efficacy comes from a study that found that active IBD patients on high dose drug regimens to control IBD flares, indicating more aggressive inflammation in this patient subset, had reduced success when administered FMT to treat recurrent *C. difficile* infection (123). In addition, IBD patients are more likely to experience intestinal flares following FMT, although the precipitating factors for these flares could be the progression of the underlying IBD condition, the recurrence of *C. difficile* or the FMT itself (124). Data on whether a microbiome transplant recipient’s intestinal inflammatory status is in an active flare phase or in maintenance phase may be crucial in understanding microbiome transplant failure or success. Currently, there are very limited clinical studies considering this factor when reporting trial results. In a small study on patients with active UC, individuals taking immunosuppressants had a stronger response rate following FMT (5 out of 11 patients) compared to UC patients not on immunosuppressants (4 out of 27 patients) (125). In the same study FMT was found to be more effective in patients (75% efficacy) who were recently diagnosed with UC compared to those with chronic disease (18% efficacy) suggesting persistent chronic inflammation may shape the intestinal microenvironment to be refractory to FMT (125).

## Section 5: Microbiome-based therapies acting on the immune system

**Figure 2:**
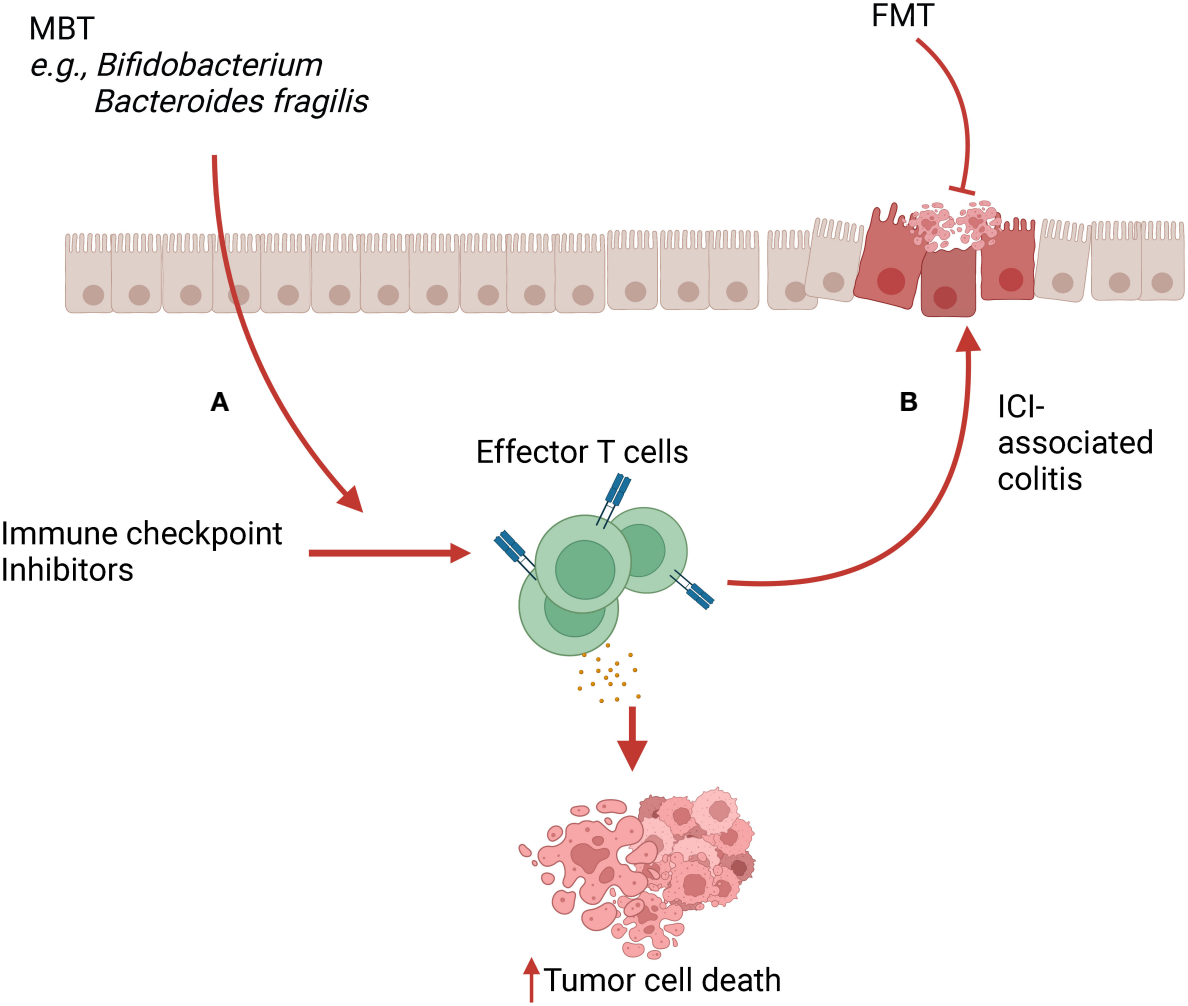
Microbiome-based therapy (MBT) in immune checkpoint inhibitor (ICI) therapy and ICI-associated colitis. **(A)** MBTs such as *Bifidobacterium* spp and *Bacteroides fragilis* cooperate with ICI, leading to increased infiltration and activation of effector T cells in the tumor microenvironment (TME). This mechanism boosts the anti-tumor activity of ICI therapy. **(B)** Activated T cells and their released cytokines induced by ICI therapy can cause off-target inflammation, leading to ICI-associated colitis. FMT can alleviate this ICI-associated colitis *via* inducing immunoregulatory properties.

Microbiome-based therapies are designed to reshape the composition of resident microbial communities and restore health. One mechanism through which these therapies elicit their beneficial properties is *via* stimulation of the immune system. Broadly, the microbiome transplant can promote immune tolerance mechanisms to decrease inflammation (**Figure 1**) or activate the immune system to enhance effector activity (**Figure 2**).

### 5.1 Microbiome-based therapies promoting immunoregulation in intestinal disorders

#### 5.1.1 IBD

Animal and clinical studies investigating microbiome-based therapies for IBD have focused on assessing whether the recipient's intestinal microbial communities and their derived metabolites are restored following treatment. Studies focused on the impact of microbiome-based therapies on the host immune response are less prevalent. In the dextran sodium sulfate (DSS)-induced colitis mouse model, FMT reduces colonic inflammation and improves mucosal immune health by inducing IL-10 expressing CD4^+^ T cells and invariant NK T cells in the colon of mice (126). Additionally, FMT reduces macrophage and neutrophil numbers in the colon and dampens antigen presentation capabilities to T cells, which is critical to controlling exaggerated inflammation (126). In agreement, a study by Wei et al. found that in DSS-induced colitis, FMT upregulates the aryl hydrocarbon receptor, IL-10, and transforming growth factor beta (TGF-β) expression in the mouse colon, providing favorable therapeutic effect in limiting DSS-induced colitis (127). Interestingly, contrary to the above studies, *Il10* ^-/-^ mice that develop spontaneous microbiome driven colitis (128) exhibit increased pro-inflammatory cytokines in the colon and exacerbated colitis following ileocolic resection and subsequent FMT administration (129), suggesting the FMT loses it therapeutic effect in the absence of IL-10. These studies suggest the prominent role of IL-10 signaling in the therapeutic effects of FMT against colitis in mice. Identifying specific immune pathways activated by the FMT will be important in determining the therapeutic outcome of the FMT.

Like FMT, live biotherapeutic bacteria have protective properties in mouse colitis models by modulating the immune response in the colon. For example, Round et al. showed that colonization of germ-free mice with *B. fragilis*, a prominent commensal bacterium, induced T_reg_ cell function and anti-inflammatory cytokine release from the T_reg_ cell population (72). This immunomodulatory effect of *B. fragilis* was driven by PSA, inducing differentiation of CD4^+^ T cells into FoxP3^+^ T_reg_ cells that produce IL-10. *B. fragilis*-derived PSA administered as a postbiotic therapy alone had an immunomodulatory effect and prevented and ameliorated TNBS-induced colitis in mice. Oral administration of a defined consortia of 17 *Clostridia* commensal bacteria also alleviated colitis in mice through T_reg_-mediated immunomodulatory effects (38). Mechanistically, these 17 *Clostridia* strains produced SCFAs that increased TGF-β concentration in the mouse colon leading to elevated colonic T_reg_ cells that expressed ICOS and produced IL-10. A follow up study further demonstrated the ability of these 17 *Clostridia* strains to produce SCFAs (73). Another defined consortium of bacteria reported by Zhu et al. also found an IL-10 associated beneficial effect. This probiotic cocktail with an equal mixture of 3 *Bifidobacterium* and 7 *Lactobacillus* sub-strains significantly reduced signs and symptoms of DSS-induced colitis and was associated with an increase of IL-10 and decrease of proinflammatory cytokines in the serum (74). The improvement in colitis was also associated with an increased expression of epithelial tight junction proteins, indicating restoration of compromised barrier functions. These observations suggest an IL-10-dependent pathway as a prominent mechanism through which microbiome transplants convey their therapeutic effect in limiting inflammation.

Commensal bacteria-derived metabolites, such as SCFAs, have also been studied for therapeutic potential in murine colitis models. In a TNBS-induced colitis model, Chen et al. found that butyrate administered through drinking water protected mice from severe colitis by suppressing proinflammatory responses in the colon and improving gut barrier integrity (130). This protective effect of butyrate was GPR109a receptor-mediated as GPR109a deficient mice completely lost butyrate-mediated protection. Several other animal studies also demonstrate protective role of SCFA against experimental colitis through boosting T_reg_ cells and downregulating pro-inflammatory cytokines in the colon (131, 132). Similarly, secondary bile acids demonstrate anti-inflammatory effects on immune cells and alleviate intestinal inflammation in a mouse model of colitis (111). Secondary bile acids derivatives, such as 3-oxoLCA and isoalloLCA can promote the differentiation of T_reg_ cells and inhibit T_H_17 cells (36, 37, 39).

Numerous clinical microbiome transplant studies report changes in transplant recipient’s immune parameters following transplantation in IBD. In a pilot study on active CD by Vaughn et al., a single colonic FMT infusion lead to an increase of T_reg_ cells in the colon at 12 weeks. However, the T_reg_ cell population increased in both FMT recipient patients who responded and failed to respond to the FMT (67). Another prospective, open-label study with 20 active UC patients showed a decrease in T_reg_ and T_H_1 cells in the colon at four weeks following a single dose of colonoscopy-delivered FMT, with no differences in T_H_2 and T_H_17 cells (68). Patients treated with Lactobacillaceae-enriched probiotic, named VSL#3, showed therapeutic benefit against active UC, compared to the placebo group. VSL#3 had an immunomodulatory effect on colonic dendritic cells (DCs). Specifically, immune profiling from rectal biopsies obtained from patients demonstrated a decrease in IL-12 and an increase in IL-10 production from the colonic DCs. Probiotics can modulate DC populations and lower the proinflammatory response in the colon, contributing to clinical improvement of UC patients (133). *Lactobacillus casei* administered rectally, but not orally, with 5-ASA therapy significantly improved clinical outcomes in UC patients, compared to 5-ASA-treatment alone (134). The clinical improvement was associated with immune changes in the colon. Biopsies from the sigmoid region of the large intestine showed decreased expression of IL-1β and TLR-4 and increased levels of IL-10 in the colon of patients who benefitted from *L. casei* probiotic. SCFAs have also been tested as a postbiotic therapy to treat IBD in patients. Luhrs and colleagues report that UC patients treated with butyrate have a significant clinical improvement compared to a non-butyrate treated group (135). Histologically, butyrate treated patients had less mucosal inflammation, reduced infiltration of neutrophils and lymphocytes in the lamina propria of the colon, associated with decreased activation of NF-κB signaling in macrophages. Other clinical studies also demonstrate immune mediated beneficial effect of SCFA in UC patients through decreasing the infiltration of neutrophils (75) and suppressing mucosal inflammation in the colon (76).

#### 5.1.2 ICI-colitis

Immune checkpoint inhibitors (ICI) work by removing the intrinsic breaks of the immune system and thereby boosting the effector functions of T cells effector function (136). One serious adverse effect of ICI use is colitis caused by an overactivated immune response, which resembles IBD and can be life-threatening. Recent studies suggest that the development of ICI-associated colitis is correlated with gut microbiome dysbiosis and restoration of the bacterial population can mitigate the ICI-associated colitis in animals and patients (137) (**Figure 2**).

In a mouse study, anti-CTLA-4 treatment was combined with DSS administration to model severe ICI-associated colitis. Oral administration of *Bifidobacterium* species rescued mice from colitis in a T_reg_ cell dependent manner without an apparent impact on the antitumor activity of anti-CTLA-4 immunotherapy (138). In a follow up study, this group found that *Bifidobacterium* enhanced mitochondrial fitness and IL-10 activity of intestinal T_reg_ cells to limit ICI-induced colitis (139). Similarly, *Lactobacillus reuteri* administration inhibits the progression of ICI-associated colitis in mice administered DSS in their drinking water and treated with anti-CTLA-4 and anti-PD-1 immunotherapy (140). The protective effect of *L. reuteri* in preventing colitis was associated with decreased infiltration of leukocytes in the intestinal tissue and decreased levels of proinflammatory cytokines such as TNF-α, IFN-γ, and IL-6 in serum, compared to non *L. reuteri*-administered mice. Additional studies have identified specific bacterial-derived metabolites with therapeutic effects against ICI-associated colitis. Mice orally administered with indole-3-carboxaldehyde (3-IAld), a microbial-derived tryptophan catabolite, had decreased disease severity, weight loss, and colonic damage compared to mice non-treated with 3-IAld (141). This protective effect of 3-IAld was AhR-signaling mediated and dependent on IL-22 cytokine production, which protected mice by inducing Reg3γ expression in the colon.

In humans, a case report of two patients with ICI-associated colitis that were refractory to steroid treatment, found FMT successfully treated the colitis (142). In one patient, the clinical improvement was associated with a substantial reduction in colonic CD8^+^ T cells with a concomitant increase in T_reg_ cells. The second patient had a decrease of all the T cell subtypes following FMT; however, the CD4^+^ T cell population was relatively unchanged compared to the CD8^+^ T cells, which was associated with the persistence of T_reg_ cells in the colonic mucosa. In another recent case study (143), a patient suffering from palatal malignant melanoma developed severe ICI-associated colitis after the third dose of anti-PD-1 treatment. After unsuccessful treatment with corticosteroids, an FMT was administered to successfully resolve colitis symptoms. The patient’s clinical outcome was associated with decreased fecal calprotectin and restoration of the congested, inflamed colonic mucosa to near normal level (143). These mucosal immune changes reported in the case reports suggest that FMT is signaling to reshape intestinal immune cell populations resulting in therapeutic effect against ICI-associated colitis. It is unknown, however, whether FMT-mediated immunoregulation during ICI treatment counteracts the anti-tumor activity of the checkpoint blockade therapy and changes cancer progression. For example, T_reg_ cells contribute to tumor growth by suppressing effector T cell functions (144). In addition, increased levels of T_reg_ cells in the tumor microenvironment are positively correlated with poor prognosis in various cancers (145, 146). As mentioned above (142), increased level of T_reg_ cells in the colon could be one of the potential mechanisms of FMT-mediated amelioration of ICI-associated colitis. Therefore, it will be important for future studies to determine whether there is any detrimental effect of FMT-driven T_reg_ cells on the capacity of ICI to limit cancer progression. Studies investigating the efficacy of microbiome-based therapies to limit ICI-associated colitis have involved a small number of patients with various types of cancers. Large-scale randomized trials are needed. A clinical trial is currently underway on the use of FMT for treating ICI-associated diarrhea or colitis in patients with genitourinary tract cancer (NCT04038619).

### 5.2 Microbiome-based therapies driving immune activation

Data from animal studies and clinical trials also indicate that the microbiome can enhance the antitumor responses of ICI (**Figure 2**). In mice, anti-CTLA-4 antibody treatment controls melanoma and colon cancer progression in specific pathogen-free (SPF) mice but not in germ-free mice. Additionally, the anti-CTLA-4 effect was compromised in SPF mice treated with broad-spectrum antibiotics supporting an active role of the bacteria (24). Further animal studies demonstrated the therapeutic role of microbiome transplants in boosting the antitumor effect of ICIs. Routy et al. found that germ-free or antibiotic-treated mice receiving FMT from cancer patients who responded to ICIs, had improved antitumor activity following anti-PD-1 treatment compared to mice receiving FMT from ICI-non-responder (147). This study also showed a correlation between the clinical benefit of ICIs with the relative abundance of *Akkermansia muciniphila*. Oral supplementation with *A. muciniphila* restored the efficacy of PD-1 blockade in non-responsive mice by increasing the recruitment of CCR9^+^ CXCR3^+^ CD4^+^ T cells into mouse tumors in an IL-12-dependent manner. This study demonstrates a clear immune-mediated role of a specific bio-therapeutic bacteria species in improving the efficacy of ICIs during cancer therapy. Tanoue et al. showed that a consortium of 11 bacterial strains obtained from healthy human donors can boost the efficacy of anti-PD-1 immunotherapy in suppressing adenocarcinoma and melanoma growth in mice by increasing the infiltration of IFN-γ^+^ CD8^+^ T cells in the tumor microenvironment (148). Interestingly, these 11 strains of bacteria are normally present in low abundance in the intestinal microbiome but could have strong potential for ICI-mediated cancer therapy if enabled to expand in the intestinal tract. Another study by Sivan et al. demonstrated that oral administration of *Bifidobacterium* enhanced the capacity of anti-PD-L1 treatment to control melanoma growth (23). This effect was mediated through activation of antigen presenting cells that enhanced CD8^+^ T cell infiltration into the tumor microenvironment. Similarly, in another mouse study, *B. fragilis* was shown to boost anti-CTLA-4 effect against tumors in mice (24). The anti-CTLA-4 anti-tumor response in germ-free or antibiotic-treated mice was rescued by oral administration of *B. fragilis*, immunization with *B. fragilis* polysaccharide, or by adoptive transfer of memory *B. fragilis*-specific T_H_1 cells. This immunostimulatory effect was mediated by an IL-12 dependent T_H_1 cell response that controlled the tumor progression. In another study of colorectal cancer and melanoma in mice, oral administration of live *Lactobacillus rhamnosus* augmented the antitumor activity of anti-PD-1 immunotherapy by triggering IFN-β production in dendritic cells with a concomitant increase of CD8^+^ T cells in the tumor microenvironment (149). These animal studies demonstrate the promise of microbiome-based therapy in supporting immune checkpoint inhibitor therapy’s antitumor effects.

Combining microbiome-based therapies with immune checkpoint inhibitors to enhance anti-cancer effects is actively being tested in the clinic with several promising results. Baruch et al. investigated the role of FMT in ICI-refractory melanoma. In this Phase I clinical trial, FMT was effective in three out of ten patients at improving the efficacy of anti-PD-1 therapy to treat metastatic melanoma. In responders, treatment with FMT was associated with a favorable immune change in tumor microenvironment, including upregulation of the IFN-γ-mediated signaling pathway, MHC-II protein expression, dendritic cell differentiation, T_H_1 cell response, and an increased infiltration of CD8^+^ T cells (150). Another study by Davar and colleagues also showed some promise using FMT for patients with refractory melanoma resistant to ICI (92). In this study fecal samples were obtained from melanoma patients who responded to ICI therapy previously and transferred to non-responsive patients. Following FMT, six out of fifteen patients clinically benefitted from FMT therapy in conjunction to anti-PD-1 treatment. Patients who responded had increased microbiome diversity, increased activation of CD8^+^ T cells, and decreased IL-8 expressing myeloid cells in tumor microenvironment (92). Beyond the two studies mentioned, several other ongoing clinical trials are investigating FMT in improving ICI therapy against various tumors, including gastrointestinal cancer (NCT04130763), renal cell carcinoma (NCT04758507) and prostate cancer (NCT04116775).

Live biotherapeutics also show promise to improve the efficacy of ICIs in human cancers. Patients with metastatic renal cell carcinoma, administered CBM588 (contained *Clostridium butyricum*) and both anti-PD-1 and anti-CTLA-4 immunotherapies had improved clinical outcome (151). The clinical improvement following CBM588 therapy was associated with an anti-tumorigenic immune change in patients. Higher level of pro-inflammatory cytokines, such as GM-CSF, IL-8, TNF-α, IFN-γ, MCP-1, IL-1RA and decreased level of T_reg_ cells were observed in peripheral blood of CBM588 plus anti-PD-1 and anti-CTLA4 group, compared to only anti-PD-1 and anti-CTLA-4 treated group. A probiotic, called EDP1503 containing *Bifidobacterium spp*, developed by Evelo Biosciences is now in Phase I/II trial for the treatment of colorectal cancer and breast cancer with ICIs (**NCT03775850**). Microbiome-derived SCFAs are also found to be associated with favorable clinical outcome to ICI therapy against human cancer. A study by Nomura et al. found that patients having high SCFA levels in blood and feces were associated with improved efficacy of anti-PD1 therapy against solid tumors (152), while in another study, patients with non-small cell lung carcinoma treated with anti-PD-1 immunotherapy showed a positive correlation between anti-PD-1 response and high levels of fecal propionate and butyrate (153). These studies do not show a direct causative link between administering SCFAs to patients and improved efficacy of ICI nor provide a mechanism of how SCFAs elicit clinical improvement in patients. Future large-scale animal and clinical studies will be needed to determine the role of postbiotics in this aspect.

### 5.3 Microbiome-based therapies tuning immune defenses against infection

#### 5.3.1 *C. difficile*

As detailed previously, the most successful implementation of FMT is in resolving *C. difficile* infection. Most animal studies investigating FMT’s mechanism of action have focused on bacteria-bacteria interactions between the FMT and *C. difficile* rather than the microbiome transplant driving host-immune changes that then lead to resolution of infection. For instance, secondary bile acid producing commensal bacteria like *Clostridium scindens* directly inhibit the growth of *C. difficile* through production of these secondary bile acids (90). However, human FMT trials have reported considerable immune changes following transplant. A pilot study found that following successful FMT for recurrent *C. difficile* infection, there was an upregulation of bile acid driven FXR-FGF signaling in the ileum, which resulted in an increase of fibroblast growth factor (FGF)-19 and decrease in FGF-21 in serum (154). The upregulation of this pathway was associated with the restoration of intestinal microbiome and secondary bile acid profile in the patient’s colon. Both secondary bile acids and FXR signaling have been linked to restoring immune tolerance; secondary bile acids *via* promotion of T_reg_ cell differentiation and FXR signaling by improving intestinal barrier integrity (36, 37, 39, 155). Therefore, restoration of secondary bile acids following FMT (86), in addition to directly inhibiting *C. difficile* growth (90, 156), may signal through the host to indirectly support resolution of *C. difficile* infection. Similarly, another study by the same group showed recurrent *C. difficile* infection patients have increased specific micro RNAs (miRNA) following FMT compared to the screening control group (157). miRNAs are short noncoding RNA molecules that can bind to complementary sequences in the 3’ UTR (3 prime untranslated region) of messenger RNAs (mRNAs), resulting in translational suppression of target genes (158). The increase of two miRNAs observed in the patient population following FMT, miR-23a and miR-150, was confirmed using a mouse model of FMT and recurrent *C. difficile* infection (157). Through target scan prediction analysis and over expression of miRNA in epithelial cells, this report found that miR-150 and miR-23a target *Il18* and *Il12b* respectively, two cytokines that promote a type-1 proinflammatory immune response. Thus, FMT driven increases in miRNA that could work to limit deleterious inflammation during *C. difficile* infection and enable inflammation-sensitive microbes from the FMT to recolonize the intestine. Immunoregulatory changes following FMT were also reported by Marie and colleagues who observed increased IL-25 levels in the colon following FMT (61). IL-25 drives type 2 immunity, which is protective following acute *C. difficile* infection in mice by skewing the host response away from a pathogenic proinflammatory response (159, 160). In the same study, the FMT suppressed the pro-inflammatory immune response and increased expression of a family of homeobox and laminin genes that promote epithelial development and homeostatic function of the colon. Further, T_H_17 cells in the peripheral blood were decreased following FMT (61). While the total T_H_17 cell populations decreased, another study reported that *C. difficile* toxin B-specific T_H_17 cells as well as toxin-A and toxin-B-specific IgG and IgA antibodies increased in the blood following successful FMT (161). The authors proposed that the enhanced *C. difficile* toxin-specific adaptive immune response could be a mechanism through which FMT works. Combined, the reported immune changes following FMT support the concept that the FMT, in addition to direct bacteria-to-bacteria interactions, could boost host immunity to clear *C. difficile* infection.

#### 5.3.2 Multidrug-resistant organisms

The success of FMT in treating recurrent *C. difficile* infection has led to the application of microbiome-based therapies to treat other bacterial infections. Multidrug-resistant organisms (MDROs) in the intestinal tract, such as vancomycin-resistant enterococci (VRE), carbapenem-resistant enterococci, methicillin-resistant *Staphylococcus aureus* (MRSA) and extended-spectrum β-lactamase producing bacteria, are resistant to more than three classes of antibiotics (162, 163). Therefore alternative treatment options are needed to combat infection originating from these organisms. In general, FMT decreases the abundance of antibiotic resistance genes in the intestinal microbiome reservoir (164). Several clinical studies have described the use of FMT for the intestinal decolonization of MDROs (165, 166). In a meta-analysis of 23 studies that included 142 patients undergoing FMT to decolonize MDROs in the intestines around 77.5% of patients cleared MDROs as determined by microbiological assessment of fecal samples and rectal swabs (167). Probiotics are also being used to prevent and treat infections caused by MDROs. For example, oral administration of two *Lactobacillus* strains (Y74 and HT121) reduced the colonization of VRE in mice colons (168). In another clinical study MRSA colonization of the gut was reduced by oral administration of *Lactobacillus rhamnosus* (169). In these examples microbiome-based therapies are targeting MDROs *via* direct bacteria to bacteria interactions that are immune independent. The mechanisms of action for the transplanted bacterial inoculum include competition for resources, production of bacteriocins, or metabolic byproducts that are toxic to the pathogenic bacteria. For example, administering an FMT or a defined bacterial consortium containing *Blautia producta* and *Clostridium bolteae* cleared persistent VRE colonization in mice colons (170, 171). Kim et al. identified that a lantibiotic produced by a specific strain of *B. producta* was necessary for clearance of VRE (172). Of note, this group observed that transplantation with the *B. producta* and *C. bolteae* consortium resolved VRE colonization even in severely immunodeficient mice (*Rag2* ^-/-^ *Il2rg* ^-/-^) further demonstrating the mechanism of action of this microbiome-based therapy was immune independent. This result contrasts with Littman et al. (114) that observed that success or failure FMT therapy was dependent on the host’s immune status. These examples illustrate that immune-independent benefits of microbiome-based therapy, which are not the focus of this review, is a rich field of study to itself (18). Understanding when microbiome-based therapies work through the immune system and when they are immune independent will be critical information as design of microbiome-based therapies become more refined and targeted.

#### 5.3.3 Viral infection

Microbiome-based therapies also can exert trans-kingdom protection against enteric and systemic viral infections. Several probiotic studies show decreased severity or duration of viral infection following probiotic administration (173, 174). Some probiotic effects are *via* non-immune-mediated mechanisms such as bacteriocin production that also have viricidal properties (175, 176) or inhibition of viral attachment to host cells (177). The microbiome also induces activation of antiviral immune defenses to poise the host to rapidly respond to invading viruses (178–182). Several probiotic formulations have been linked to enhanced type I and II IFN signaling and natural killer cell activity to limit viral infection (183–185). This is further supported by mouse studies that isolated and transplanted live bio-therapeutic bacterial species that activated type I IFN-mediated antiviral defense pathways resulting in improved host survival against enteric or systemic viral infection (181, 182). Steed et al. reported that an FMT could protect microbiome-depleted mice from influenza virus infection and went on to identify that a specific postbiotic, desaminotyrosine, produced by *Clostridum orbiscindens* through flavonoid metabolism could be administered to enhance type I IFN pathways in the lung and protect against lethal influenza virus challenge (186). Microbiome-based therapies can also enhance adaptive antiviral immunity. Several probiotics can enhance secretory IgA antibody production to limit enteric viral entry (187–189). Meanwhile administration of the postbiotic PSA derived from *B. fragilis* limited fatal herpes simplex encephalitis. This therapeutic effect was driven by PSA tuning down the T cell response through increasing IL-10 production (190). Indeed, probiotics have even been proposed as part of a treatment regimen to correct the dysbiosis associated with SARS-CoV-2 infection (191, 192).

## 6 Concluding remarks

The promise of using microbiome-based therapies to treat disease has as much potential to improve human life as when the first heart and liver transplant surgeries were performed in the 1960-70’s. However, the same microbiome transplant inoculum often has a heterogenous clinical outcome when administered to multiple patients (193). Long term engraftment in the recipients can be determined by multiple factors such as diet, genetics, or the pre-existing microbiome (194–197). Therefore, in the same vein as fifty years ago when the rules of organ donor matching and immunosuppression medication were systematically teased apart through careful, basic scientific research, the mechanisms determining microbiome transplantation success need to be identified to harness the potential power of this nascent field.

## Author contributions

ZA researched and wrote the manuscript. JM designed the figure and provided edits feedback. MA reviewed the manuscript and provided edits and feedback. All authors contributed to the article and approved the submitted version.
